# Data Mining and Pattern Recognition Models for Identifying Inherited Diseases: Challenges and Implications

**DOI:** 10.3389/fgene.2016.00136

**Published:** 2016-08-10

**Authors:** Lahiru Iddamalgoda, Partha S. Das, Achala Aponso, Vijayaraghava S. Sundararajan, Prashanth Suravajhala, Jayaraman K. Valadi

**Affiliations:** ^1^Department of Computing, Informatics Institute of Technology, University of WestminsterColombo, Sri Lanka; ^2^Department of Microbiology, Bioinformatics Infrastructure Facility, Vidyasagar UniversityMidnapore, India; ^3^Bioinformatics, Bioclues OrganizationHyderabad, India; ^4^Environmental Health Institute, National Environment Agency, SingaporeSingapore; ^5^Molecular Biology and Genetics, Quantitative Genetics and Genomics, Aarhus UniversityTjele, Denmark; ^6^Bioinformatics, Bioinformatics OrganizationHudson, MA, USA

**Keywords:** inherited diseases, data mining, machine learning, single nucleotide polymorphism, protein-protein interaction

## Abstract

Data mining and pattern recognition methods reveal interesting findings in genetic studies, especially on how the genetic makeup is associated with inherited diseases. Although researchers have proposed various data mining models for biomedical approaches, there remains a challenge in accurately prioritizing the single nucleotide polymorphisms (SNP) associated with the disease. In this commentary, we review the state-of-art data mining and pattern recognition models for identifying inherited diseases and deliberate the need of binary classification- and scoring-based prioritization methods in determining causal variants. While we discuss the pros and cons associated with these methods known, we argue that the gene prioritization methods and the protein interaction (PPI) methods in conjunction with the K nearest neighbors' could be used in accurately categorizing the genetic factors in disease causation.

## Introduction

Many human diseases that have a causative association with genetic components are called as inherited diseases. Recent advances have significantly improved our understanding on diseases and inherited factors that play an important role in the disease paradigm (Schrodi et al., 2014). While it is a challenging task to identify the variants associated with inherited diseases through wet-lab based techniques, there is a need to find the causal effects of genetic changes associated with inherited diseases such as Autism, Schizophrenia, Bipolar disorder, etc. However, due to the complexity of the human genome, information from traditional methods such as human pedigree analysis has been in demand. In humans, as crosses cannot be performed due to ethical reasons, genealogical records need to be scrutinized to distinguish autosomal diseases from other forms of inherited diseases like X-linked diseases. Conversely, animals have been employed as models to ascertain factors linking to such diseases and causal mutations. From these studies, the pedigree or genealogy trees are interpreted to understand concurrent pairs of phenotypes for the diseasome studies. Such phenotypic studies would further allow us to understand the inheritance patterns of a disease associated with genetic polymorphism.

In the recent-past, there is a great deal of information outlying the genes associated with polymorphisms in relation to single nucleotide polymorphisms (SNP), genetic variants, multi nucleotide polymorphisms (MNP), quantitative trait loci (QTL), gene ontology (GO), and protein-protein interactions (PPI) or association and transcriptomic datasets coming from RNA-Seq data (Costa et al., 2013; Schrodi et al., 2014). Nevertheless, the genetic variation can be best seen with the intragenic/intronic regions or those that are non-coding or non-regulatory in nature. As most of the sequences associated with non-coding or non-regulatory regions, especially miRNAs, are highly conserved, it would be difficult to find the SNPs associated with them and their principal component part of diseasome studies is debated. Although the root cause for such genetic models could be studied by patterns associated with these polymorphisms, there remains a challenge on how these models are essential for understanding different data types.

## Performance of the SNP based approaches for identifying inherited diseases

**Figure 1:**
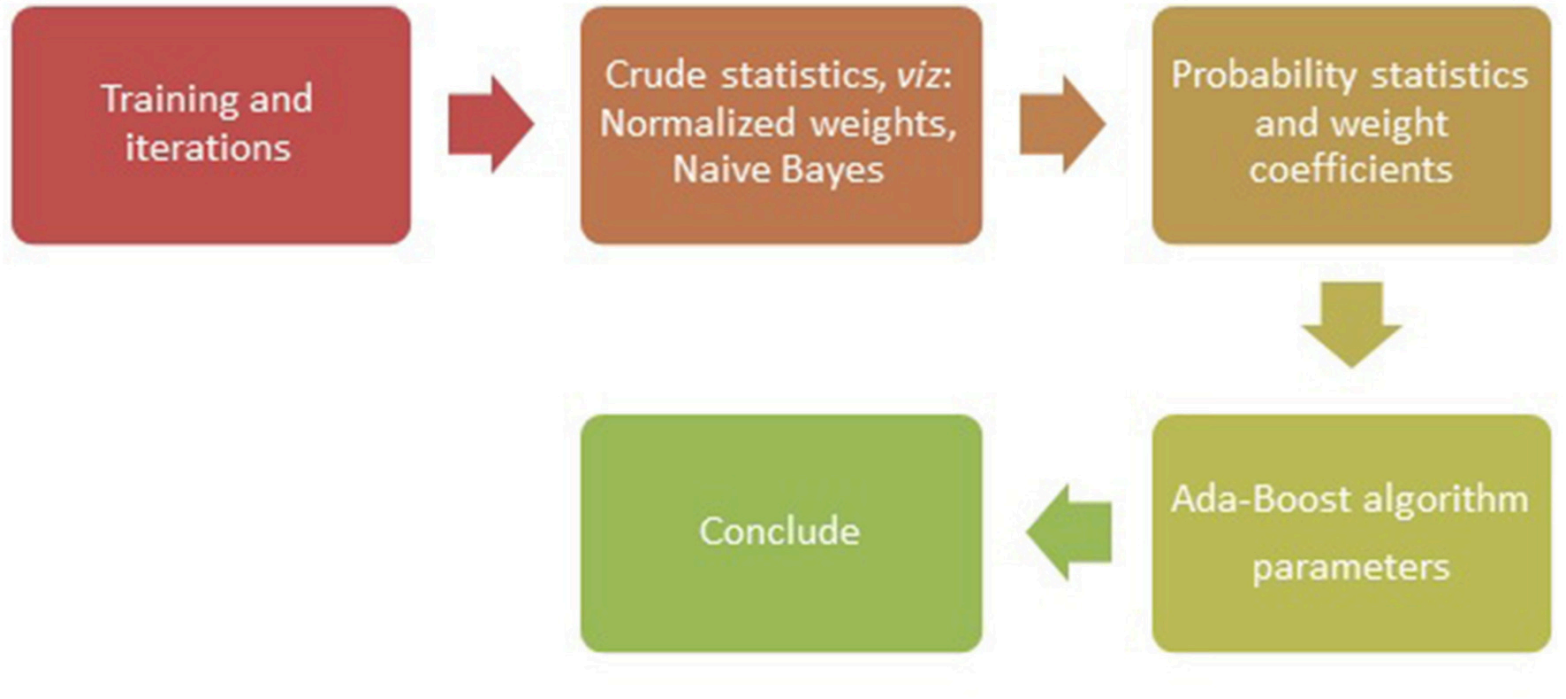
**Flow chart of the Ada-Boost Algorithm**.

With the SNPs as genetic variants (Jiaxin et al., 2010), traditional classification methods and novel data mining techniques were explored to show effectiveness of different algorithms in identifying the disease association (Jiang et al., 2007). The SNPs located within protein coding regions can be further categorized into synonymous and non-synonymous SNPs (nsSNPs). A synonymous SNP does not alter the protein sequence, whereas the non-synonymous substitutions potentially affect protein function that may result in diseases (Yates and Sternberg, 2013). In methods used for classification and machine learning, the nsSNPs have been known to serve as better candidates, for example in studies involving binary classification with ensemble learning approach (Breiman, 1999). The methods such as AdaBoost (Figure 1), Random forest, L2boosting, stochastic gradient regression are known to fall short of classification methods such as decision tree and support vector machines (Jiaxin et al., 2010; Benso et al., 2013).

**Figure 2:**
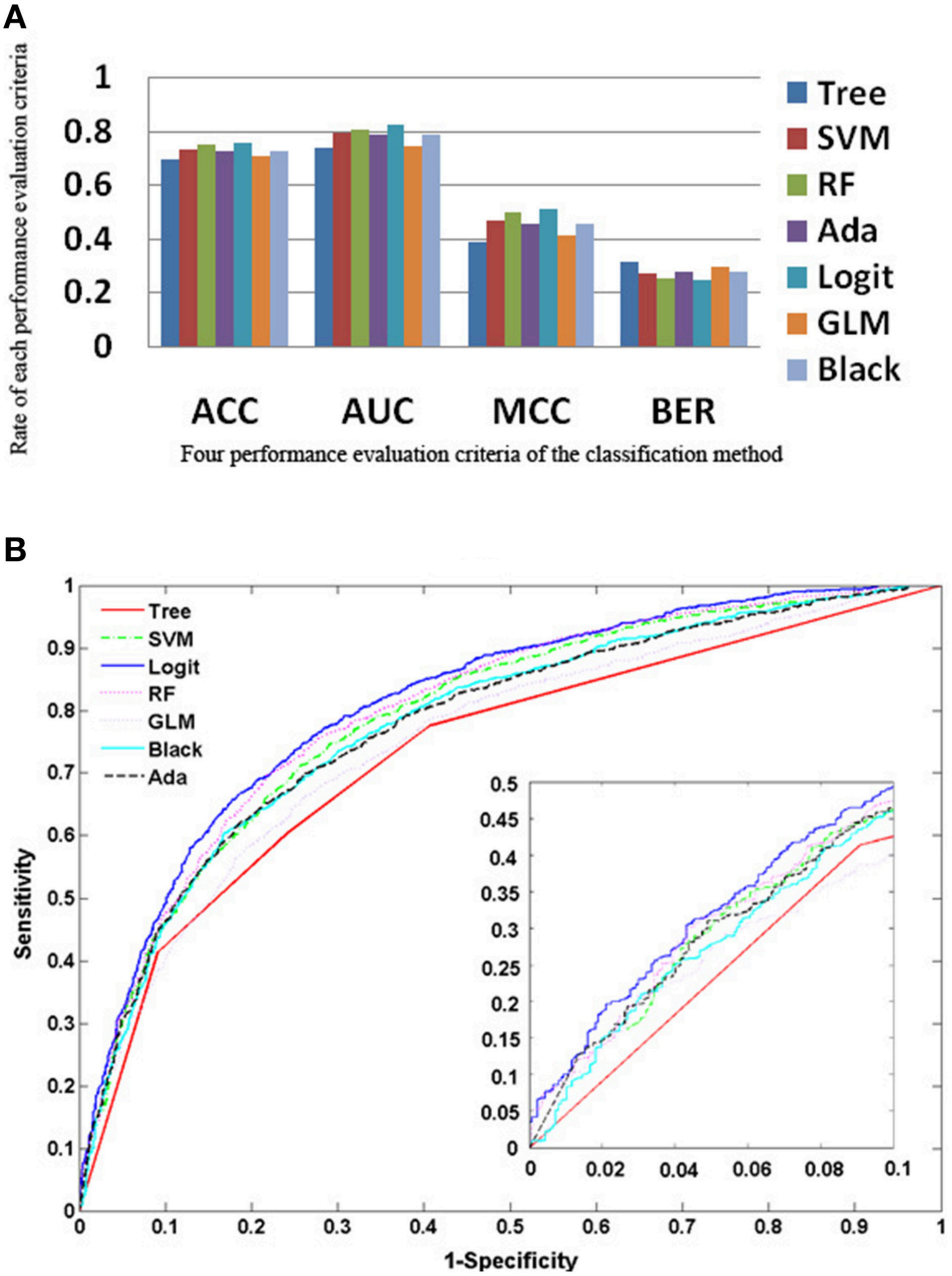
**Performance of the approaches as explained from the methods (A) Rate of performance evaluation comparison of the each classification methods; the accuracy of the prediction (ACC); the area under the receiver operating characteristic (ROC) curve (AUC); the balanced error rate (BER) and the Matthews' correlation coefficient (MCC). (B)** Performance of the 7 approaches based on the true positive rate vs. false positive rate.

The five ensemble learning approaches and two classification methods are briefly tabulated in Table 1. Essentially, the following three categories of data are integrated to identify disease-causing SNPs of statistical significance: (a) annotations of nsSNPs extracted from the Swiss-Prot database (Consortium, 2010), (b) annotation of the protein families and structural domains extracted from Pfam database (Finn et al., 2006), and (c) a domain-domain interaction network obtained from the DOMINE (Raghavachari et al., 2008) and the InterDom database (Ng et al., 2003). From our preliminary observations, when we test all the classifiers against the above data, they seem to perform well in disease causing nsSNPs against regular nsSNPs. However, when comparing only four pre-set evaluation criteria, we find them to have significant differences from the random situations (Figure 2). The performance of approaches in identifying SNPs associated with diseases is measured by accuracy of the prediction (ACC), proportion of correctly classified cases (Horn et al., 2003), the area under receiver operating characteristic (ROC) curve (also called AUC), understating the prediction power of a given classification method, the balanced error rate (BER), and Matthew's correlation coefficient (MCC) which represents the prediction power under a certain decision threshold considering the biased and unbiased samples. Generally, smaller the BER, larger are the ACC and MCC. These methods have been reviewed elsewhere and are in agreement with the datum that Logit boost algorithm is the best method (Jiaxin et al., 2010). Results of the decision tree are distinguished more from other ensemble classifiers; BER of the decision tree is higher than other classifiers. The performance wise arrangement of the classifiers are L2boosting < stochastic gradient regression < SVM < Adaboost < random forest tree < logitboosts (Jiaxin et al., 2010). These methods for prioritizing candidates are based on the integrated use of two-sequence conservation features and methods such as domain-domain interaction networks. The bioinformatics based methods such as PolyPhen (Ramensky et al., 2002), SIFT (Ng and Henikoff, 2003), KBAC (Liu and Leal, 2010), and MSRV (Jiang et al., 2007) along with binary classification methods provide limited information in prioritizing disease-associated nsSNPs when compared to multiple sequence alignment (MSA) methods (Rui and Jiaxin, 2011) which extracts conserved protein sequences underlying the mutation.

**Table 1:** **Comparison of the Ensemble learning approaches**.

**Methods**	**Description**	**Features**
AdaBoost	Adaboosting (adaptive boosting) is one of the most popular boosting algorithms. This algorithm is characterized by its adaptive changes to fit sample weights during the boosting process, according to the weighted classification error identified from the last training (Vapnik, 1991).	Advantage of the decision tree as the basic “weak” classifier (Jiaxin et al., 2010).
LogitBoost	Logiboost is an improved version of the AdaBoost algorithm. The main difference between Adaboost and Logiboost is that Logiboost puts confidence on the binomial log-likelihood as a loss function, more likely in binary classification than the exponential basis underlying the Adaboost algorithms.	Compared with Adaboost, LogitBoost is found to be more effective in case of noisy data, easier to implement and does not require tuning and model or kernel selection like neural networks or support vector machines. LogitBoost can work with Logit models, decision stump, or decision trees (Friedman et al., 2000; Jiaxin et al., 2010).
Random Forest trees	Random forest is a boosting method implemented for voting most popular tree after many classification trees have been grown. In large data sets, the method shows outstanding efficiency.	This method has several advantages, like few parameters to be adjusted, no over-fitting problem, fast computational speed, and a strong ability of anti-noise characteristics. In addition, Random forests have a built-in method to estimate the importance of features. This method is usefully to prioritize the features by their importance and reduce the feature set in order to improve the computational complexity (Jiaxin et al., 2010).
L2boosting	L2boosting is a gradient boosting algorithm for optimizing arbitrary loss functions where component based linear models are made use of based learners. It has shown better performance compared to decision stumps (tree with two terminal nodes) and other more common competitors, particularly when the predictor space is multi-dimensional.	In addition, L2boosting works well with both regression and classification problems. It shows comparably better performance for classification related problems like LogitBoost (Jiaxin et al., 2010).
Stochastic gradient regression	Stochastic gradient is a regression prediction method. This method uses regression tree as a base learner. The optimization of the gradient descent, stochastic gradient regression utilizes the pseudo-residuals resulting from negative gradient of loss function to set up iterative regression tree.	This algorithm randomly selects part of the pseudo-residual to make regression tree instead of the whole pseudo-residuals. This model can be a linear combination of some regression trees (Jiaxin et al., 2010).
Support Vector Machine	Support Vector Machine (SVM), also known as “Support Vector Network” is a machine learning method for binary classification problems, although implementations of multi-class SVMs exist to map input vectors to a multi-dimensional feature space. A linear decision environment is built with special properties ensuring high generalization ability of a machine learning approach.	The idea behind the support vector network has been extensively implemented in biology with some method for the restricted case where training data can be separated without errors, further extending this result to non-separable training data (Cortes and Vapnik, 1995).
Decision trees	This method applies to scenarios in which specific decision alternatives cannot be predicted with high level of confidence. It is a hierarchical modeling system for supervised learning where local regions are identified by a sequence of recursive splits in few steps. A tree here is composed of decision nodes and terminal leaves. The trees can be of various types like univariate trees, classification trees, regression trees etc. When making a decision, a lot of different factors are taken as inputs, the decision tree uses its own feature selection strategy to select only those useful for classification (Breiman et al., 1999; Alpaydin, 2014).	Decision tree solves complex decisional problems having significant uncertainty(Safavian and Landgrebe, 1991).

To overcome this limitation, we introduced methods that integrate conservation properties of amino acids and domains harbored calculated association score (Rui and Jiaxin, 2011). A brief description is given below.

### Calculation of similarity scores between nsSNPs

The purpose of using following equations is to measure the similarity scores between a single pair of nsSNPs, first getting an nsSNP and the corresponding amino acid substitution occurring at a certain position. The probability of occurrence of the original amino acid (*P*_*org*_) is calculated at a similar position of the protein super family. For this purpose, the Pfam database is used to extract the multiple sequence alignment (MSA) of the query protein to find the number of occurrences of the original amino acid at the corresponding position of the alignment and then to divide the number of occurrences by the number of proteins in the alignment (Rui and Jiaxin, 2011).

First Equation corresponds to probability of occurrence of the original amino acid (*P*org).

$$Sim_{org}(a,b)=1-\left|\;p_{org}(a)-p_{org}(b)\;\right|$$

Second Equation corresponds to probability of occurrence of the substituted amino acid (*P*_*sub*_).

$$Sim_{sub}\;(a,b)=1-\left|\;p_{sub}(a)-p_{sub}(b)\;\right|$$

Third Equation corresponds to calculation of diffusion kernel of the domain-domain interaction network

$${\text{Sim}}_{\text{DDI}}(\text{a},\text{b})={\text{K}}_{\text{DDI}}(\text{a},\text{b}),$$

### Prioritization of candidate nsSNPs

After calculating the single pairwise similarity measure of nsSNPs, the prioritization of a set of candidate nsSNPs is done by “guilt-by-association principle” (Altshuler et al., 2000). The association score for a candidate nsSNPs as the mean similarity value between the nsSNP and all seed nsSNPs is calculated using

$$A(c)=\frac{1}{S(d)}\sum_{s\;\in \;S(d)}Sim(c,s),$$

where c is a candidate nsSNP, *A(c)* is the association score and *S(d)* the set of seed nsSNPs from query disease *d*.

### Integrating multiple ranks

The multiple ranking lists obtained from guilt-by-association principle is applied to each data sources. Here, an altered Stouffer's Z-score method is provided to integrate the ranks and to obtain a single ranking list.

$${\text{Z}}_{\text{i}}^{\left(\text{K}\right)}=\Phi^{-1}\left(1-\frac{r_{i}^{\left(k\right)}+0.5}{\max(r_{i}^{\left(k\right)})+1}\right)$$

The integrated Z-score is made by adding up their corresponding Z-scores as following equation. Finally, they are re-ranked according to the integrated Z-scores in decreasing order to obtain the integrated ranking list.

$$Z_{i}^{\left(k\right)}=\sum_{k\;=\;1}^{m}\frac{z_{i}^{\left(k\right)}}{\sqrt{m}}$$

We argue that there are certain limitations in this approach, as it is a choice for extracting conserved protein domains using the Pfam database. To overcome the problem of identifying the variants, few other sequence alignment tools such as BLAST or PSIBLAST can be used to extract sequence conservation features (Altschul et al., 1997) which highlight the mutations in genome regions such as transcriptional-factor binding sites or promoter regions (Jiaxin et al., 2010; Rui and Jiaxin, 2011). While a binary classification solution such as Logitboost is found to be a more accurate classification algorithm, it is fully dependent on the multiple sequence alignment. However, combining the multiple sequence analysis and domain-domain interaction method is a better method to identify nsSNPs associated with diseases. Further enhancement of domain-domain interaction models would allow us to evaluate the functional similarity between two genes and their products. These data sources contain gene expression profile, gene ontology annotations, PPI.

## Combination of SNP methods attributing to inherited diseases

**Figure 3:**
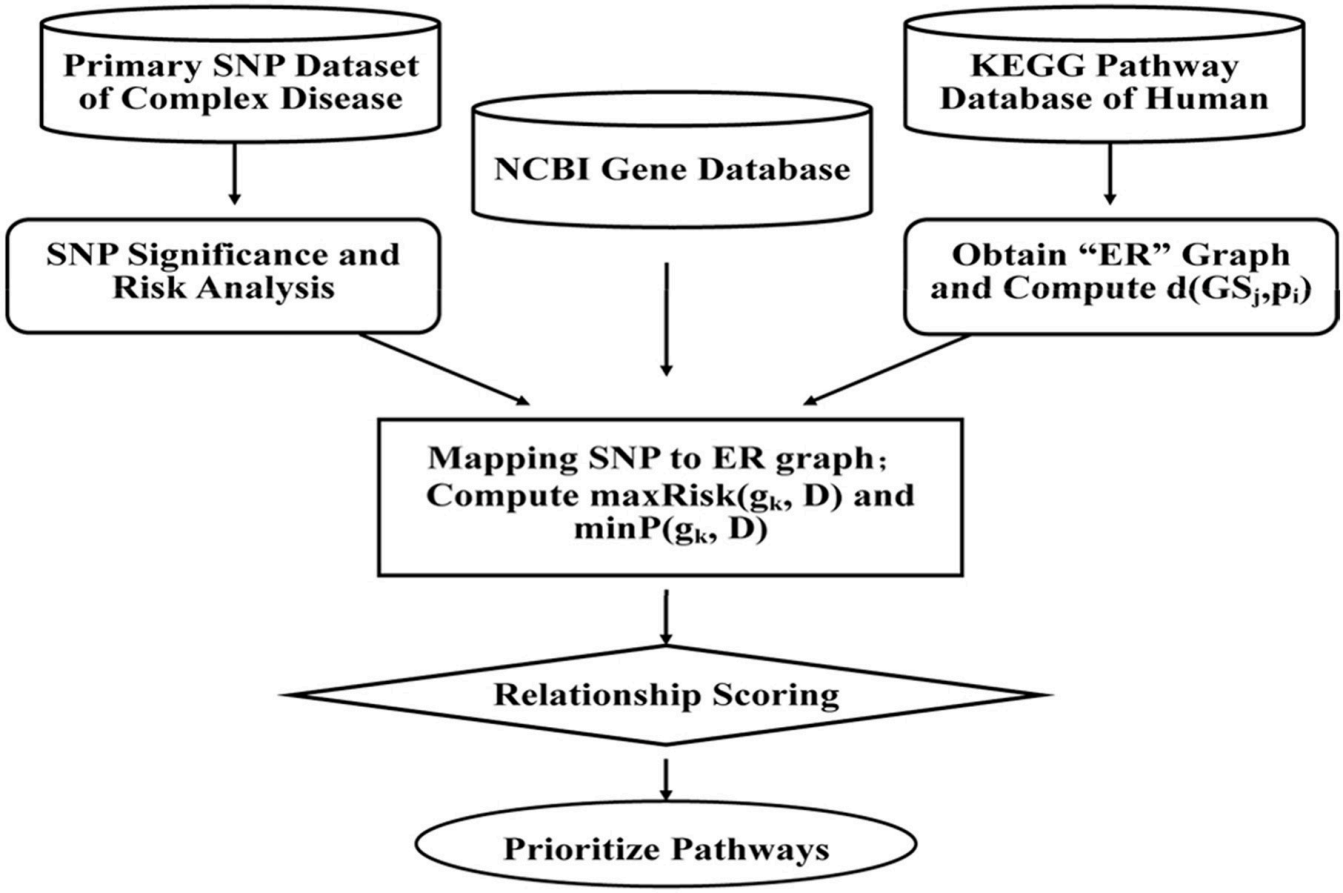
**A SNP pathway based Association Method (SPAM): First step is SNP significance analysis and the corresponding risk calculation**. Data of case and control for each and risk ratio is calculated from relationships between the SNP and complex diseases. The second step is reconstruction of KEGG pathway and analysis of the reconstructed network attribution. As per the format, KEGG pathway represents a network where a node represents metabolite and one edge represents some enzyme or a gene cluster. The third step involves screening of SNPs and mapping of SNPs to reconstructed network (Hoh and Ott, 2003). The fourth step is calculation of the two integrated measurements of RS scoring and prioritizing the pathway.

With SNPs and other clinical conditions contributing to a wide range of inherited diseases (Fiaschi et al., 2009), a general framework has been proposed to find variants for pre-eclampsia, a progressive disorder that occurs during pregnancy and soon after the birth, affecting both the mother and the baby (Roberts et al., 1989). Mutated risk genes, genetic, and environmental factors are thought to be of key importance in such diseases (Risch and Merikangas, 1996; Liangcai et al., 2008). Further methods have been proposed to analyze risk pathway of the bipolar disorder (BD; Hirschfeld et al., 2003). Keeping in view of the fact that detection of associations between human genetic variant and their phenotypic involvement is a significant challenge in understanding genetic basis of inherited diseases in humans (Wu et al., 2014), various methods, viz. ID3 (Breiman et al., 1999), ADTree (Freund and Mason, 1999), and C4.5 (Quinlan, 1993) have been recognized. Most of the current systems, however, predict associations between nsSNPs and diseases based on features obtained from only protein sequences and/or structure information, and do not provide details about which specific disease is associated with nsSNPs. Further, to evaluate combination of methodologies, analysis of the disease association of the SNPs and environmental factors in the KEGG pathways can be done through which we could represent the SNP networks of molecular wiring diagrams, in addition to mapping genes to the reaction and interactions (Kanehisa and Goto, 2000; Liangcai et al., 2008). There are few major steps of calculating disease risks which focus on genetic factors involved in the relationship between multiple genes and the diseases, but also the metabolic environment factors between genes and pathways. This can be further achieved by calculating the two RS scores and by prioritizing the pathways. The RS measures are integrated according to condition-dependent theory, and the association between the biological pathways and the complex disease is established through sorts of genes. The measurements algorithm, viz. a SNP Pathway based Association Method (SPAM) is briefed as follows (also see Figure 3) (Liangcai et al., 2008).

$$RS\;(D,P_{i})=\sum_{j\;=\;1}^{N}\left\{d(GS_{j},p_{i})*\frac{1}{M}\sum_{\underset{g_{k}\;\in \;GS_{j}}{k\;=\;1}}\max\;Risk(g_{k},D)\right\}$$

Or

$$RS\;(D,P_{i})=\sum_{j\;=\;1}^{N}\left\{d(GS_{j},p_{i})*\frac{1}{M}\sum_{\underset{g_{k}\;\in \;GS_{j}}{k\;=\;1}}\left[1-\min p(g_{k},D)\right]\;\right\}$$

Here, *RS* (*D*, *p*_*i*_) is the relationship scoring between pathway *p*_*i*_ and the disease D, N is the number of gene clusters on pathway *p*_*i*_ and *d*(*GS*_*j*_, *p*_*i*_) reveals the complexity of gene cluster *GS*_*j*_ on pathway *p*_*i*_. m is the number of genes with *p* < 0.05. *M* is the count of all genes on pathway *p*_*i*_.

While comparing our method (SNPs analysis) with earlier stated methods, we argue that other methods are effective only in detecting partially known genetic or unknown genetic bases and are used for analyzing queried protein sequences. To overcome this limitation, PSI-BLAST (Altschul et al., 1997) or COBALT (Papadopoulos and Agarwala, 2007) enables feature selection methods. Family history related data can additionally be used to infer performance of the proposed solution (Fiaschi et al., 2009), in addition to feature selection entities such as transcriptional and promoter regions (Liangcai et al., 2008; Wu et al., 2014). Whereas the combination methods explain more originality of how SNPs specific to diseases can be analyzed, KEGG pathways are useful to scrutinize other environment factors with SNP data. The phenotype data associated with diseases and its threshold values are used to transform categorical data into Boolean ones (Fiaschi et al., 2009) which helps in machine learning. However, it must be done judiciously as selection may significantly affect the accuracy of the solution. Wu et al. (2014) argue that Canberra distance (Emran and Ye, 2001) algorithm is the best to calculate distance between pairwise nsSNPs but in some cases, the same distance may be inferred between two similar pairs in the dataset as it is hard to classify or to associate with the particular diseases.

Given the numbers of diseased gene prioritization generated by wet lab techniques serve as a major impediment in human genetics, bringing a multi-faceted approach for diagnostics and treatment (Arrais and Oliveira, 2010) for diseases such as autism (Kim et al., 2000), schizophrenia (Jingchun et al., 2008) and diabetes should be strategically prioritized. That said, the similarity between all strategies is the use of “guilt-by-association” concept where the most relevant candidates will be the ones that are similar to the genes already known to be linked to the biological process of interest. Graph based models for gene-disease prioritization consider biomedical terms such as genes, pathways, homologies, ontologies, gene expression data and literature in ascertaining the model (Arrais and Oliveira, 2010). The previous gene-disease prioritization models have been built using gene related concepts to construct the questions over biomedical databases and to create ranked list of genes. Following this, from MeSH (Xu and Li, 2006), the statistical and knowledge based combining data from gene ontology and MeSH (Raghavachari et al., 2008) apart from probabilistic methods, viz. Hyper-Induced Topic Search (HITS; Kleinberg, 1999) and PageRank (Page et al., 1999) have been very useful. However, the above methods lagged by annotation coverage and tend for large biases.

## PPI networks and inherited diseases

**Figure 4:**
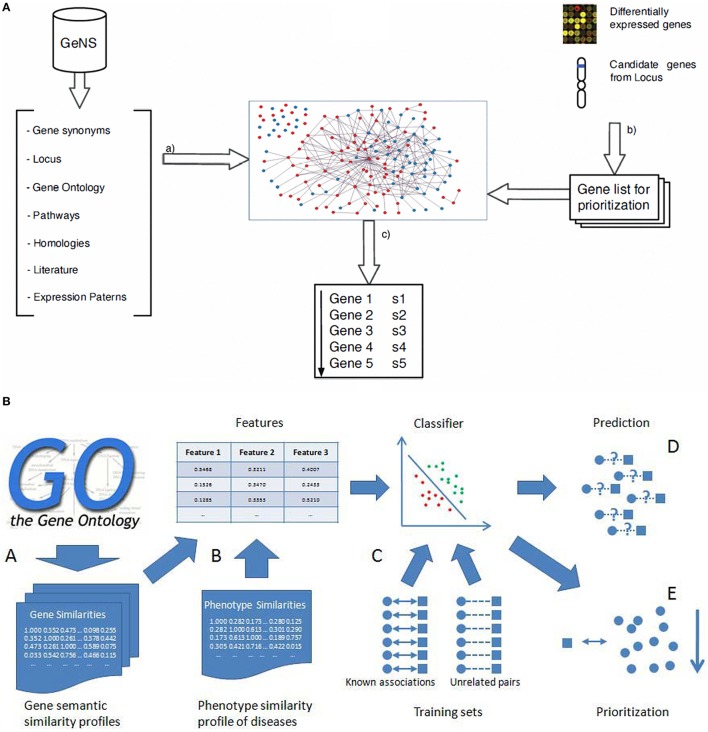
**Gene prioritization methods. (A)** (a) Entities and their relations are retrieved from the source databases which are then filtered, sorted and disambiguated to create the graph. From gene expression studies or positional studies or GO, a list of genes are obtained for prioritization (Arrais and Oliveira, 2010) (b) Calculation of the gene semantic similarity scores to obtain the three gene similarity profiles; **(B)** Combining the three gene similarity profiles and the phenotype similarity profile to calculate three dimensional features for classification; (C) Training classifier using the known associations and sufficient unrelated gene-disease pairs as training sets; (D) Prediction of disease-gene associations; (E) Prioritization of candidate genes (He and Jiang, 2012).

The availability of human genome-wide PPI data has opened a wide outlook for discovering inherited disease genes by studying topological features in PPI networks (Xu and Li, 2006). Keeping in view of the fact that studies on proteins and their interactions are important to understand their dynamic roles for identification of inherited, and to a certain extent rare immunological disorders, mapping the disease specific genes (for example genes related to schizophrenia) into the whole human interaction network and then the extraction of related sub networks can throw light on the cellular mechanisms and biological processes related to the inherited disease (Jingchun et al., 2008). The sequence based features have previously been exploited and found that in many cases there are significant differences between genes responsible for human hereditary disease and those not known to be involved in diseases (Xu and Li, 2006). Genes associated with a human disease preferentially interacted with other disease-causing genes, suggesting that heritable disease-genes might share some topological features in the PPIs network when compared to the non-diseased genes (Gandhi et al., 2006). We further argue that similar genes are obtained with human PPI datasets from Online Predicted Human Integration Database (OPID; Brown and Jurisica, 2005) employing K-nearest neighbor (KNN) algorithm for classification. The KNN algorithm is a simple and yet a powerful non parametric classification algorithm (Franke et al., 2006) with an effective performance. With rapid enhancement in quantity and quality of human interaction and phenotypic data, the performance and utility of this approach to detect novel disease-genes should improve further as we come to the end of the post-genomic era. We have earlier proposed a classification scoring method to validate the interaction mapping between such proteins and calculated the total reliability score (TRS) using machine learning algorithms (Suravajhala and Sundararajan, 2012). The accuracy of six point classification model was found to be 81.08% on multilayer perception of neural network which, if used based on such approaches, could have identified causal SNPs toward development of molecular markers. In the recent-past, the PPI database is also used as a knowledge base with a set of known disease-related genes that is utilized with linkage analysis in prioritizing the best candidates. From our previous discussions, we argue that the main advantage of the probabilistic knowledge model is that it reduces the prioritization error by 6% when compared to already published methods. These studies based on the relatedness to known diseases or closely related disease processes, however, remain a challenge in prioritizing loner or isolated genes with no known relationships between two nodes in the network. The solution for prioritizing loosely connected disease genes with other genes has previously been proposed by Fang et al. (Fang et al., 2014) (Figure 4) which uses network diffusion and rank concordance (NDRC). In addition, they found that genes related to complex diseases are divided into several modules associated with different disease phenotypes. First, they built the network without removing the insignificant genes from the network, while the second one was based on the Diffusion Rank (DR) algorithm (Yang et al., 2007). The NDRC simulates the heat diffusion process where information flows from the known disease genes of related disease and propagates over the PPI network with noisy data as a problem to prioritize disease genes (Li et al., 2009; Wang et al., 2009; Fang et al., 2014). Multiple kernels learning (KML) and N dimensional order statistic (NDOS) methods were found to be handling noisy data effectively. Two strategies are relatively known, one to search for a kernel that would best represent all the information available using a convex optimization method, known as semi-definite programming (SDP; Lanckriet et al., 2004; De Bie et al., 2007) which simultaneously optimizes parameters for one-class SVM and tests genes ranked by the one-class SVM. In the second strategy, all test genes were first ranked using one-class SVM with an individual data source and then, N-dimensional order statistics was used to combine these rank lists into one rank list (Lanckriet et al., 2004). For gene ontology and sequence data, we argue that the kernel with higher weight may not have much influence in discerning the SNPs. Different machine learning algorithms as described are used for prioritizing SNPs and proven to be effective especially SVMs with higher performance than the random forest. A comparative study was carried out by Tranchevent et al. (2010) on prioritizing tools. The authors recommended genes to diseases (G2D) as a good tool for providing an ordered list of candidate genes in the peak regions (Perez-Iratxeta et al., 2002). In conclusion, SVM can be applied to the gene prioritizing process with ontology association as it encompasses enrichment based feature selection processes in identification of inherited diseases (He and Jiang, 2012; Xie et al., 2012). Nevertheless, feature selection method and integrating more data sources like semantic and phenotype similarity profiling between diseases and genes could build better prediction ability.

## Conclusions

Most of the inherited diseases are caused by SNP variants and research findings on causal SNPs are becoming prominent. During the last few years, the multitude of research showcasing SNPs and other methodologies has come up in identifying the candidates for inherited diseases. From this commentary, we reason out that identification of SNPs has generally been considered as a binary classification problem, although, there are a host of methods, viz. support vector machine tools, random forest methods, gene prioritization methods that are used for scoring and calculating genes truly associated with the inherited diseases. Among the classifiers, KNN is known to be the most best performing algorithm for the analysis of inherited diseases based on PPI network. As human PPIs grow in post-genomic era, a promising source for discovering such disease genes could herald a need to understand the rigorous algorithms behind such approaches. As researchers use different cross validation methods to prove accuracy, efficiency, and the appropriation, a conceptualized framework for identification of inherited diseases would be promising. With deluge of human genomic data containing SNPs and PPI, there remains this challenge.

## Author contributions

LI and AA made the initial survey and validation of studies, PD, VS, and PS discussed and mentored the graduate students toward their virtual project, JV advised and guided the review, PS and JV proofread the manuscript.

### Conflict of interest statement

The authors declare that the research was conducted in the absence of any commercial or financial relationships that could be construed as a potential conflict of interest.
